# Perspectives on Care for Late-Stage Parkinson's Disease

**DOI:** 10.1155/2021/9475026

**Published:** 2021-03-15

**Authors:** Kristina Rosqvist, Marianne Kylberg, Charlotte Löfqvist, Anette Schrag, Per Odin, Susanne Iwarsson

**Affiliations:** ^1^Lund University, Faculty of Medicine, Department of Clinical Sciences Lund, Neurology, Lund, Sweden; ^2^Lund University, Faculty of Medicine, Department of Health Sciences, Lund, Sweden; ^3^University College London, Queen Square Institute of Neurology, London, UK; ^4^Department of Neurology, Skåne University Hospital, Lund, Sweden

## Abstract

In the late stage of Parkinson's disease (PD), there is an increasing disease burden not only for the patients but also for their informal caregivers and the health and social services systems. The aim of this study was to explore experiences of late-stage PD patients' and their informal caregivers' satisfaction with care and support, in order to better understand how they perceive the treatment and care they receive. This qualitative substudy was part of the longitudinal European multicentre Care of Late Stage Parkinsonism (CLaSP) project. Individual semistructured interviews were conducted with patients (*n* = 11) and informal caregivers (*n* = 9) in Sweden. Data were analysed through the content analysis technique. The final analyses generated one main category: “We are trying to get by both with and without the formal care” and five subcategories: “Availability of health care is important for managing symptoms and everyday life”; “Dependence on others and scheduled days form everyday life”; “There is a wish to get adequate help when it is needed”; “Mixed feelings on future housing and respite care”; and “Family responsibility and loyalty for a functioning everyday life”. Having regular contact with PD-specialised health care was perceived as important. Greater access to physiotherapy was wished for. Maintaining autonomy was perceived as important by patients, in both home health care and a future residential care setting. Responsibility and loyalty between spouses and support from children enabled everyday life to carry on at home, indicating a vulnerability for those without an informal caregiver. The results suggest that regular access to PD-specialised health care is important and that a specialised and multidisciplinary approach to the management of PD symptomatology is likely necessary. Non-PD-specialised staff in home health care and residential care facilities should regularly be given opportunities to obtain PD-specific education and information.

## 1. Introduction

Parkinson's disease (PD) is a progressive disorder, and in spite of several therapeutic advances since the start of levodopa therapy in the 1960s [1–4], there are still no evident disease-modifying therapies. It is, therefore, most important to optimize symptomatic treatment and care throughout the course of the disease, not least in the late stage.

In late-stage PD, i.e., Hoehn and Yahr (HY) [5] stages IV and V in the medication “on” state, motor and nonmotor symptoms (NMS) are pronounced [6–9], and the patients are dependent on assistance in activities of daily living (ADL) [6]. Consequently, there is a high demand on the health care system and the municipality-based home health care and social services as well as an increasing burden for informal family caregivers [10]. However, there is little known how late-stage PD patients and their informal caregivers perceive the care and treatment they receive, in different health care systems in Europe [11–13].

A North American study showed that close to 60% of PD patients reported satisfaction with their overall care [14]. Similarly, in a recent quantitative study that included the current sample, 59% of late-stage PD patients and 59% of their informal caregivers reported satisfaction with their overall care. Furthermore, the results indicated that satisfaction with care in late-stage PD is negatively associated with depressive symptoms and positively associated with independence in ADL. Informal caregivers' satisfaction with support was positively associated with patients' HY stage as well as with caregivers' quality of life (QoL) [15].

To date, there have been few studies focusing specifically on the late stage of PD [7], a patient group that is likely to become larger in the future due to increased life expectancy and improved health care [6, 16]. Therefore, there is a need to better understand how patients in the late stage of PD and their informal caregivers perceive how their needs are met by health care and social services.

Through qualitative methodology, it is possible to achieve a deeper understanding of patients' and their informal caregivers' subjective needs and perceptions of how health care and social services can meet their needs.

The aim of this study was to explore experiences of late-stage PD patients' and their informal caregivers' satisfaction with care and support, in order to better understand how they perceive the treatment and care they receive.

## 2. Materials and Methods

### 2.1. Study Context

This qualitative substudy was part of the European longitudinal multicenter project, Care of Late Stage Parkinsonism (CLaSP), the largest study on late-stage PD to date, in which seven centres in six countries participated [17]. Inclusion criteria for the CLaSP project were HY stages IV and V in the medication “on” state and/or having a substantial need of help with ADL (defined here as ≤50% on the Schwab and England Scale) [18], as well as having been diagnosed with idiopathic PD for at least seven years. Exclusion criteria were cognitive symptoms that started before the PD diagnosis as well as symptomatic Parkinsonism, such as drug-induced Parkinsonism or normal pressure hydrocephalus. Three centres of the CLaSP project participated in the qualitative substudy.

### 2.2. Participants and Recruitment

The study was designed to interview approximately 20 participants at each centre, aiming for approximately ten interviews with patients and ten with informal caregivers. Participants for qualitative interviews were provided from the Swedish sample of the CLaSP project (*n* = 107) [15, 17], recruited from the southern region of Sweden through neurology departments and the municipality-based home health care and social services system. Interviewees were recruited consecutively among the participants of the main study, according to a strategic sampling procedure [19], striving for heterogeneity regarding age, gender, type of housing, and disease severity (HY stage IV or V). A special effort was made to include dyads of patient and spouse, which resulted in seven complete dyads. All individuals who were invited to take part in the qualitative substudy accepted participation. All participants had previously received information about the CLaSP project.

Twenty individuals participated: 11 of them were patients and nine were informal caregivers. All patients except for one were in HY stage IV; 9 (82%) lived at home and 2 (18%) in a residential care facility. Three (27%) of the patients were women. The median (min–max) patient age was 83 (64–89) years. Among the informal caregivers, 7 (78%) were women; all were the spouse except for one who was a daughter (Table 1).

### 2.3. Procedure

An interview guide with semistructured, open-ended questions was constructed and targeted the subject of personal and perceived needs regarding both formal and informal care in relation to PD and how these needs were met or unmet by the health care and social services. The interview guide further included areas such as thoughts on and information about moving to a residential care facility and what kind of care or services that were envisioned if the disease would worsen. The interview guide included four sets of slightly varying interview questions, depending on whether the patient lived at home or in a residential care facility.

The interviews were conducted during 2015 and 2016, in the homes of the participants at separate visits to the baseline, quantitative data collection of the CLaSP project. During the interviews, no other person than the interviewee and the interviewer was present in the room, in order for each interviewee to be able to speak freely, without being affected, e.g., by the presence of the spouse. Before starting the interview, the interviewer repeated the purpose of the CLaSP study to the interviewee. The interviews lasted between 20 and 60 minutes. The interviews were audio-recorded and transcribed verbatim. Fifteen of the interviews as well as the main transcription of the interviews were performed by the first author. Five of the interviews and four transcriptions were performed by a research nurse.

### 2.4. Setting

The Swedish health care and social services system includes specialised medicine and primary health care, provided by county councils and private enterprises, as well as municipally controlled health care and social services. Governed by the Health Care Act and Social Services Act, municipalities provide home health care, social services, short-term care, and needs assessed assisted living/residential care facilities. In addition, there are private health care and social services providers.

### 2.5. Ethical Considerations

Ethical considerations mainly concerned the frailty and vulnerability of the patients and the often stressful situation of their informal caregivers regarding the care for their family members. Accordingly, during the recruitment of participants and data collection, the researchers were highly responsive to the situation and needs of the participants. The anonymity and confidentiality of the participants were maintained throughout the research process.

The study was approved by the Regional Ethical Review Board in Lund, Sweden (JPND HC-559-002). Written informed consent was obtained by the participants.

### 2.6. Data Analysis

Data were analysed according to the content analysis technique, using a directed content analysis [20]. Initially, the transcripts were read through by the first and second authors (KR, MK) to obtain an overview of the material as a whole. This inductive reading [19] helped to understand the content of the interviews and to find patterns in the data in order to form a framework for the deductive coding process in the NVivo software (NVivo 12). The framework consisted of two main categories and six subcategories used for sorting data from the interviews into the subcategories. The first and second authors carried out the coding individually and as an interactive process. Joint discussion on coded data related to the different subcategories resulted in adjustments of the subcategories and coded material. The final framework consisted of one main category and five subcategories covering the experiences of formal and informal care for patients and their informal caregivers who live in the late stage of PD. Finally, a validation of the subcategories and the main category was carried out by three of the coauthors (SI, SL, and PO), by reading two interviews each and giving input on how these interviews were represented in and contributed to the findings.

## 3. Results

**Figure 1:**
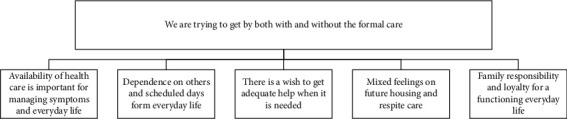
Main category with five subcategories which illustrate the patients' and informal caregivers' experiences of health and social care in late-stage PD in Sweden.

Both the patients and the informal caregivers described their experiences of how living in the late stage of PD affected their everyday life, containing positive as well as negative experiences of health care and social services in relation to frequency, staff competence, and quality of care. Over time, the patients' opportunities for activity and participation had decreased, and even if they strived for independence, the need for formal and informal assistance increased. Informal care was important for the patients and characterised by responsibility and loyalty within the family. Even if assisting a spouse/family member sometimes could be difficult, the informal caregivers talked about the importance of still being together. These experiences were categorised into one main category and five subcategories (Figure 1). Two different kinds of experiences related to health care and social services were found. The first kind was the experiences directly linked to the services received (subcategories 1 and 2), while the second kind was emotional experiences where loyalty within the family was expressed (subcategories 3–5).

### 3.1. We Are Trying to Get by Both with and without the Formal Care

#### 3.1.1. Availability of Health Care Is Important for Managing Symptoms and Everyday Life

Generally, patients and their informal caregivers reported that the contact with Parkinson-specialised neurologists and PD nurses worked well. However, there were some patients who had experiences of not being listened to when discussing medications or other treatments. Even though most of the patients and their informal caregivers appreciated their annual visit to the PD specialist/neurologist, they experienced that the visits mostly focused on rapid assessments of walking ability and the medication. Most of them would instead rather have had a more thorough follow-up of the previous visits, for example, on how the medicine had worked, nonmotor problems, and possible side effects. One patient expressed severe depressive symptoms and had sometimes experienced suicidal thoughts and indicated a need for psychological well-being as well as the situation at large to be addressed. Some patients expressed an interest in advanced therapy (i.e., deep brain stimulation or advanced dopaminergic therapy, such as levodopa-carbidopa intestinal gel and apomorphine subcutaneous infusion) [21], which they felt excluded from due to age and disease duration. Another PD-related problem for one of the informal caregivers was the discussion of driving with the patient, where she felt that the PD specialist/neurologist had put all the responsibility on her. Most of the patients and the informal caregivers would have appreciated more frequent contact with the PD specialist/neurologist than the annual visit offered at present. It was often difficult to reach the PD specialist/neurologist between the visits, although the PD nurse could often be contacted for support if questions arose between the visits to the PD specialist/neurologist. Some patients and informal caregivers expressed that it was difficult to receive the health care they were entitled to from the health care system.

“Once a year it is, so there are long intervals in between. Before there were two visits, though it seemed they thought we could extend it. Because I can always telephone.” (Patient)

Knowledge about PD seemed to be fundamental for an experience of good care, and contacts with nonspecialised health care institutions often became problematic due to insufficient PD-specific knowledge. Mistakes were often made, not least concerning the medication. This impacted on confidence with the care provided, from both the patients' and the informal caregivers' points of view.

Almost all patients expressed that they wanted more physiotherapy and physical training. The lack of physiotherapy, especially PD-specialised physiotherapy, was repeatedly mentioned. Many had at some point trained with a physiotherapist, either in a health care setting, in a gym, or at home, and had appreciated these opportunities. However, these were typically described as time-limited, whereas many wished for ongoing physical training. One of the patients who had trained with a physiotherapist on a regular basis was convinced that he would have been in a worse condition if he had not had this training. The reported reasons for not continuing with the physical training varied among the patients; some reported that the training had ended after a certain time period and others that the training had ended due to their functional decline. In order to have physiotherapy and physical training, the patients and informal caregivers often had to take responsibility for arranging this themselves, for example, by contacting the primary care or driving to a training facility. However, to some of the patients and informal caregivers, it was not clear what training they could expect or whom to contact.

Another important factor for managing everyday life was the availability of assistive devices and opportunities for housing adaptations. All the patients used assistive devices for mobility, provided by physiotherapists or occupational therapists. There were also examples of patients who used devices for getting in and out of bed and for personal care. Some of the patients and informal caregivers had received housing adaptations such as the installation of automatic door openers, grab bars in the bathroom, or removed thresholds between rooms, to facilitate everyday life activities. However, a few of the patients and informal caregivers had not received any information on how to apply for housing adaptations and the involvement of occupational therapists in the application process and, therefore, had paid for everything by themselves. In some cases, informal caregivers themselves had taken action to improve the home and to get various devices to facilitate for the patient, either because it was not available from the municipality or because they were not aware of what could be provided. In spite of housing adaptations, patients sometimes felt that further modifications would be valuable.

#### 3.1.2. Dependence on Others and Scheduled Days Form Everyday Life

Patients and informal caregivers reported having since long adapted everyday life to the disease. Most patients talked about how their performance and level of energy varied during the day and that everything they undertook generally took much longer than previously. Some patients had developed strategies to be able to maintain activities and to avoid stressful situations by carefully thinking through various obstacles that could arise and finding solutions to them (e.g., freezing of gait or falling). Others described how they had been forced to give up activities, which resulted in increased isolation and being tied to the home.

“If I get rigid it becomes difficult, everything takes time. Much more time than it did before. I could never have become a firefighter, because I would never have kept up with the pace.” (Patient)

Medication intake, help with dressing and hygiene, toilet visits, and transfers between different rooms in the home and help with meals at predetermined times were the main factors that controlled everyday life. Most of the patients tried to manage as much as they could by themselves or together with their informal caregivers, but for those who were dependent on home health care, predetermined time points affected their degree of independence and self-determination. Informal caregivers also described that the patient's need for home health care controlled the days. While the informal caregivers appreciated the help the home health care and social services provided, it also constituted an intrusion into privacy that could sometimes be difficult to relate to.

“The home health care will come at 8 o'clock, 12 o'clock, 4 o'clock, and 8 o'clock again in the evening. And in between, I get to help NN.” (Informal caregiver)

Patients and informal caregivers often expressed disappointment that they had limited possibilities to get out of the home and to be outdoors. The informal caregivers described limitations when it came to performing activities on their own and maintaining relationships with friends, for both the patients and themselves. For informal caregivers who lived together with the patient, loss of control could be stressful, such as when they had to rely on social services or when the patient would be alone during the time they were away. A stressful situation for children who had an active caregiving role was to be able to balance the needs of the patient with their own need of time for family and work. Both patients and informal caregivers described how they received help from their children and occasionally also from siblings and neighbours to make everyday life work. Despite this, the spouses most often carried the heaviest load.

Due to the disease's impact, some of the patients and their informal caregivers had adjusted how they shared the household work between them. This had resulted in an increased burden for the informal caregivers. The most common reason for these changes was fear of the patient falling. At the same time, there was a disappointment among the patients that they could not manage themselves anymore. Few informal caregivers dared to leave the patient alone at home and, therefore, chose activities they could do together, which were adapted to the patient's abilities to participate. When leaving the home together, there would sometimes be a concern that the patient would fall.

“As he started to fall… it started affecting the gait... then it immediately became more difficult. Before we used to work together in the garden.” (Informal caregiver)

#### 3.1.3. There Is a Wish to Get Adequate Help When It Is Needed

The competence and continuity of health care staff were highlighted as important for getting adequate and high-quality care. Large staff turnover in the home health care and lack of competent staff in residential care facilities sometimes resulted in miscommunication between staff about, for instance, the medication that was to be given at predetermined times. Knowledge about PD and how it affects the everyday life of the patient was expressed by both patients and informal caregivers as essential for the staff members' understanding of when and what type of help was needed, e.g., the importance of a correctly given medication.

Even though the patients and the informal caregivers appreciated the help from the home health care, some wished for a greater understanding of the need for personal integrity as well as the chance to influence the schedule for home health care services. Predetermined time points for toilet visits and for when going to bed were difficult to deal with, even though patients as well as informal caregivers expressed that they understood the challenges with planning home health care services in advance.

The nights were in some cases perceived as a major problem, for instance, when the patient needed help to get to the toilet. Therefore, some of the informal caregivers wished for an opportunity to sleep at night and not having to be attentive to the patient's needs at all times. In some cases, there was a strong wish from the informal caregivers to get a break, with the main aim to get some undisturbed sleep.

“Last night I have not slept more than two hours at the time, maybe just one... you have to make sure you get to sleep for another little while, so, it will get better again….” (Informal caregiver)

Patients often wanted to do as much as possible themselves in ADL but experienced that the home health care staff often took over too quickly due to limited time, for instance, during mealtime or in transfer situations in a residential care facility. The informal caregivers highlighted the importance of letting the patient take an active part to the best of his or her ability in different situations as well as including the patient in an activity or a situation and not talking over his or her head.

“More staff is needed, it's as simple as that, and that the staff is taught to maybe talk TO the patient, not above the person's head.” (Daughter to a patient in a residential care facility)

“*Because it's nice to be by yourself too. And it's not nice with people running in and* out*….*” (Patient)

For patients living by themselves, the home health care and social services staff also became important from a social perspective, giving the patient someone to talk with. Some patients expressed a fear of being left alone, without having someone who could help when it was needed. This was a concern mainly for patients who lived by themselves, who could, for instance, fear being left lying on the floor after a fall in the shower. For those who lived with their spouse, it was usually the spouse who provided assistance in unforeseen situations.

*“Of course there comes a day when I can no longer, then one has to accept the circumstances and ask for help ....”* (Informal caregiver)

Generally, patients and informal caregivers wished for more individualized solutions when it comes to home health care and support in the home. When there were night-time visits from the home health care, these could constitute stress on both the patient and the informal caregiver, for instance, when the staff speak so loudly that the informal caregiver is disturbed. Therefore, flexibility and the possibility of contacting the home health care services when needed would have been desirable.

Being able to receive home health care even when visiting one's summer house was greatly appreciated and facilitated everyday life, for those in that situation. The possibility of pressing the alarm when needed was perceived as a security, while at the same time there was sometimes an uncertainty whether the alarm would work in all situations or whether those responding to an alarm would be able to help in the situation the patient was in, for instance, after a fall.

#### 3.1.4. Mixed Feelings on Future Housing and Respite Care

The patients' and informal caregivers' thoughts on moving to another dwelling, residential care facility, or service accommodation were related to a limit when the current dwelling was no longer perceived as suitable. Some of the patients together with their spouse had already early in the course of the disease taken the step to relocate, to be able to more easily cope with everyday activities, while others chose to stay in the dwelling where they had lived for many years.

Although there are help and support from the municipalities when it comes to residential care facilities, patients as well as informal caregivers described how they would prefer to stay in their present home for as long as possible. If or when moving would become necessary, the focus would be on more accessible accommodation, to make it easier for them to get out to participate in various activities, such as going to the supermarket. To be able to decide by oneself what to do and when to do, it was expressed as an important aspect of self-determination. Having to move to a residential care facility because the current situation no longer functioned was viewed in varying ways by both the patients and the informal caregivers. For some, the opportunity to move when they wanted it themselves was important, while for others there was a concern that they would be given a room without personal touch and with reduced possibilities to live based on one's own habits. Among the informal caregivers, there was a concern that the residential care facility staff would not see the patient as a person with his or her own habits or interests, forced into doing things that he or she did not like or in a way that was not appreciated. Based on earlier experiences with stays in hospitals and other care establishments, there was a fear that the knowledge about PD would be limited and that the care for the patient, for instance, concerning the medication, would be suboptimal. The patients had varying knowledge of what types of residential care facilities that the municipality offered and how to obtain information about this. The patients' prior experiences of residential care, often based on experiences related to their relatives or friends, affected their view on moving. While many expressed a fear of becoming institutionalized and not receiving a good quality of care, some saw opportunities to get help when needed and increased social contact. Generally, both patients and informal caregivers expressed a desire to carry on at home for as long as possible.

“No, I hope not ... I hope not, I want to try to manage here at home ....” (Informal caregiver)

“It would be a service accommodation, that help is close so to speak, help with the things one can't handle” (Patient)

Because patients wished to remain at home, some of the informal caregivers felt guilty when thinking about or raising the issue of the patient temporarily moving to a short-term or respite care facility. While the informal caregiver could see possible benefits for themselves with the patient's relocation to another residence, they also raised the issue of loneliness, as they, despite all the work, were good company for each other. For the informal caregivers who did not live together with the patient, the starting point to discuss a move to a residential care facility was clearer, when it became obvious that the support provided by the home health care and social services was not sufficient.

One reason why informal caregivers were doubtful about respite or short-term accommodation was fear that the patient would not receive the right care and treatment. In this context, there were further concerns that healthcare professionals not familiar with the patient's situation and lacking PD-specific knowledge would make temporary changes to the patient's medication that would interfere with the PD medication and worsen the PD symptomatology.

#### 3.1.5. Family Responsibility and Loyalty for a Functioning Everyday Life

The patients and their informal caregivers all expressed a sense of responsibility and loyalty to each other as well as a sense of belonging together. Several of them had lived together for many years and neither could nor wished to be separated from each other, as they valued being together.

“I always think that he would have done the same for me, if it were me.” (Informal caregiver)

“We have been married for over 60 years, so we know each other well, yes, so one is very happy to do this. Because we still love each other even though we are old. ” (Informal caregiver)

However, there were times when the patients were aware of or felt that they were a burden to their spouse, who had to give up his or her own activities to stay at home and take care of or watch over them. Some of the patients described that they had experienced irritation and anger from their spouses. The informal caregivers expressed that they had the patient in mind at all times and that they felt a need to watch over the patient, since there was constantly a fear of him or her falling or getting into other difficulties. An awareness was expressed from several of the patients and informal caregivers that the patient would not have been able to remain living at home without the informal caregiver.

The informal care provided by children was appreciated by both patients and their spouses. There was an understanding but also a disappointment that adult children could not always be there to help, because they were busy with their own lives, families, and jobs. Even though patients and their spouses did not expect regular assistance from their children, they reported that the children were often available when needed. Common tasks carried out by the children were helping out with shopping or driving the patient to a doctor's appointment, which was described as a feeling of security by patients and spouses. There were also examples of unevenly distributed assistance given by children, where one sibling carried a heavy daily workload while another only came to visit from time to time. In cases where children had moved to another city, the spouse often had to take on greater responsibility as the couple did not always want to burden friends and acquaintances.

“That you do not bother too much, you do not want that.” (Patient)

*“Yes, it is my wife who is, she has the greatest burden (gets moved).”* (Patient)

All the informal caregivers described themselves as the “engine,” who keeps everyday life running for the patient to be able to remain at home. They controlled the medication and physiotherapy appointments and were the contact person with the health care and social services systems. As the disease progressed, a couple had often lost contact with friends. For informal caregivers who were still working, in addition to responsibility for informal care of the patient, there could also be a desire to care for their own elderly parents or a wish to help out with the grandchildren, resulting in multiple workloads for themselves.

Some of the informal caregivers were aware of the importance of having the time of their own to be able to cope and continue supporting the patient. However, PD had become a part of life for both the patient and themselves, which they had adapted to. Despite this, attempts were made to participate in activities together, such as visiting friends and family or traveling, for as long as possible.

*“Before, you felt free, more free that is, now you always have this in mind, that you have to think of (NN, the patient).”* (Informal caregiver)

## 4. Discussion

The findings explore how late-stage PD patients and their informal caregivers experience the care and support they receive through the health care and social services system in Sweden, where personal met and unmet needs in relation to formal and informal care were expressed. This study follows our recently published study on satisfaction with care in late-stage PD [15], with qualitative informative perspectives in addition to the quantitative data of the first publication. The main result, the patients' and informal caregivers' experiences on formal and informal care for patients in late-stage PD, is reflected by the main category: “We are trying to get by both with and without the formal care,” which contained five subcategories: “Availability of health care is important for managing symptoms and everyday life;” “Dependence on others and scheduled days form everyday life;” “There is a wish to get adequate help when it is needed;” “Mixed feelings on future housing and respite care;” and “Family responsibility and loyalty for a functioning everyday life.” The results provide new insight into how late-stage PD patients and their informal caregivers in Sweden perceive their situation with regard to the treatment and care they receive. Both patients and informal caregivers considered specialist care with PD-specialised staff to be important. Most of them were not content with a yearly visit, but wished for more regular visits, including therapy modification and follow-up with their PD specialist/neurologist. This is in line with previous research indicating that PD-specialised care is essential for patient satisfaction [14, 22]. As a large proportion (60–80%) of the late-stage PD sample were still followed by a PD specialist [15, 23], this represents new insight on how access to a PD-specific team is perceived among patients and informal caregivers in late-stage PD. Access to regular physiotherapy was strongly wished for by the majority of the participants. Many of them had earlier during the disease trained with a physiotherapist, though, with advancing disease and subsequent increasing immobility, few still had regular physiotherapy. In spite of that, almost half of the total Swedish CLaSP sample had had contact with a physiotherapist, occupational therapist, or speech and language therapist within the past three months [15]. Regular physiotherapy and physical training should probably be prioritized in the care for late-stage PD, as the motor symptomatology in the late stage is pronounced and gait and balance severely affected [6, 8].

As >80% of the late-stage PD patients in Sweden received professional home health care [15], most of the patients and informal caregivers had extensive experience of home health care and social services, which in Sweden are provided by the municipalities. All the participants of the current sample had home health care and social services and their everyday life revolved around the predetermined schedule of the home health care visits during the day. Although appreciated and needed by the patients, this also constituted limited autonomy and an intrusion into privacy, which affected both the patients and their spouses. In addition to the practical aspects of home health care, patients living alone sometimes expressed an additional benefit of home health care, where they had the opportunity to have someone to talk with, feeling less lonely. This may consist of an additional benefit for the patient, as it has been indicated that living alone and loneliness are associated with a more rapid motor decline in community-dwelling older adults [24].

Enhancing the possibility for late-stage PD patients to actively take part in the planning and scheduling of their everyday care may, therefore, add a sense of participation and autonomy within the boundaries of the disease, in spite of the large practical need of the services provided. In doing this, the self-efficacy of the patient may be strengthened and greater life satisfaction experienced. Self-efficacy has been shown to be associated with life satisfaction in PD in general as well as in late-stage PD [25], and life satisfaction is markedly worsened by disease progression [25, 26]. This is in line with the results of the CLaSP qualitative substudy carried out at the London centre, where it was indicated that positive patient support in the home may help patients maintain a degree of normality and identity [27]. Furthermore, previous PD research showed that patient-perceived involvement in therapy decisions is associated with greater satisfaction and QoL [28].

For the informal caregivers, everyday life highly revolved around their partner's disease and their own life was heavily limited by the needs of the patient. Recent PD research focuses increasingly on the needs of the informal caregivers [10], whose situation should be considered and supported by the health care system. This is in line with research on frail older people in general, where recognizing and supporting the informal caregivers is concluded as essential to support people to be able to continue living at home [29, 30].

The patients' thoughts on future living and respite care consisted mainly of preconceptions of negative and pessimistic character, based on the experiences of others, often earlier in time. Both patients and informal caregivers expressed a wish to carry on with everyday life together for as long as possible, in their own home. Therefore, it is important for health care and social services to provide optimal solutions to enable this, which is, moreover, of economic value for the municipalities and in line with the current practice within the municipalities. There was, however, an awareness among the patients that there may be a time when they can no longer manage in their own homes. During such a situation, patients feared this would mean they would be given a room without their personal touch and wished for independent solutions where it was possible, maintaining their own habits and interests. Like with home health care, maintaining autonomy where possible seems to be crucial for good QoL. These results are in line with the results of the qualitative substudy at the London centre, where the patients indicated uncertainty and little planning for the future and expressed that moving to a residential care facility was not desired, although viewed as eventually necessary [27]. This further indicates that late-stage PD patients' perceptions of their situation may be similar, in spite of various national contexts.

Autonomy is a fundamental human right, including older people living in residential care. In such facilities, dependency needs should be tended to, while personal autonomy is maintained [31]. Increased autonomy has been pointed out as the most important factor for well-being in a residential care facility setting [32] and has been linked to independence in ADL [33]. Having the possibility of making certain choices for oneself has been linked to higher degrees of perceived autonomy; for example, being able to choose when to go to bed was rated as important by 83% of the participants in a Belgian study investigating autonomy among residential care facility residents [33]. This was expressed also in the present study, as those who heavily depended on home health care and had predetermined time points for assistance when going to bed perceived that it highly affected their autonomy.

In the total Swedish CLaSP sample, there was no significant difference in satisfaction with care among those with or without a partner [15]. The loyalty and joy of companionship and of still being together outweighed the burden of the disease for the informal caregivers in the present study. There was an awareness among both patients and spouses that it was because of the efforts of the informal caregiver/spouse that they were able to keep up everyday life at home in the late stage of the disease.

In congruence with the quantitative results [15], the present qualitative findings showed that the majority of the patients and informal caregivers were satisfied with their overall care, while pointing out areas and issues in health care and social services where they perceived that improvements could be made. In the present study, the patients and their informal caregivers have highlighted this, from a late-stage PD perspective.

### 4.1. Strengths, Limitations, and Future Perspectives

Qualitative research methods allow gaining insight into how late-stage PD patients and their informal caregivers experience their needs in regard to health care and social services, which is not provided by quantitative methods. In this study, we performed interviews with late-stage PD patients and their informal caregivers to get valuable insight into how they perceive the care that they receive in relation to their needs. This provides new knowledge of the situation for patients with late-stage PD and their informal caregivers, which has so far been very limited. In the late stage of the disease, patients are to various degrees heavily affected by the PD symptomatology, such as fatigue, dysarthria, and cognitive decline, which may all have affected the possibility of participating. These aspects sometimes provided challenges in the interview situation, which had to be adapted to each individual's ability to participate, resulting in different depth and length of the interviews.

Diversity sampling [19] was strived for, which implies that patients in various stages of the disease, with various ages, genders, dwellings, and family constellations, were included, in order to enrichen the content of the data gathered for this study. The younger and healthier individuals of the sample were generally able to provide more information than older individuals who were more severely affected by the disease. However, in spite of these obstacles, precious aspects of how the care in late-stage PD was perceived were collected from all participants and data saturation was reached. The informal caregivers were generally able to speak more freely than the patients, though there was some variation in how much information each interview generated.

It should be kept in mind that the current study was based on the Swedish subsample of the CLaSP project. As the qualitative substudy was carried out nationally at each participating centre, there will likely be differences based on the variety in how the health care and social services systems are constructed in each country. This may provide insight into what is perceived as desirable and well-functioning among various national systems. In Sweden, a vast majority of the patients in late-stage PD receive municipality-based home health care and social services. This may not be the case in other countries, where more of the responsibility may lay on the informal caregivers as well as services offered through private providers. Future studies may attempt to compare late-stage PD patients' experiences throughout various health care and social services settings, as there is an ambition to continue one step further with cross-national analyses within the CLaSP project.

Hoping to continue improving treatment and care in late-stage PD, in order to enhance QoL, could in the future include increased use of advanced pharmacological therapy [21], as well as other pharmacological and nonpharmacological interventions [34–36].

The researchers' preexisting understanding affects the analyses in qualitative methodology, where previous knowledge could affect the way the data were interpreted. The current analysis was carried out mainly by two of the authors: one with significant knowledge in the field of PD and the other with broad experience and expertise in the qualitative methodology used in research on ageing and health, which meant the data were viewed from different perspectives. The validation stage, which involved three senior researchers representing different disciplines (neurology, occupational therapy, and gerontology), can be considered a notable strength.

One should bear in mind that a large proportion of the interviewees in this study were of “the grateful generation, which does not complain” (born in the late 1920s and 1930s), which may imply that the needs of this population may be greater than what was expressed in the present study. Reasons for this could be gratefulness, not to show vulnerability or even pride.

It was clear from the quantitative analyses on the total Swedish sample that patients and informal caregivers may view the situation they share differently, as it was only in 36% of the cases that both the patient and the informal caregiver responded that they were both satisfied with their overall care and support [15]. This indicates that attention from health and social support systems should be given to both the patient and the informal caregiver [37]. Through qualitative data, additional aspects can provide deeper information and a valuable understanding of what may lay behind the data collected in quantitative surveys. The present study provides insight into which specific areas are important for patient satisfaction with care and informal caregiver satisfaction with support in late-stage PD. Therefore, it provides additional perspectives to the results based on quantitative data and constitutes part of a base for further research on late-stage PD as well as in work striving to improve health care and social services for this patient group.

## 5. Conclusions

Having regular contact with PD-specialised health care staff was perceived as important by all the late-stage PD patients and informal caregivers included in this study. Access to regular physiotherapy was further wished for. Maintaining autonomy was perceived as important for the patients, both when it comes to home health care and social services and in future residential care settings. Responsibility and loyalty between spouses and support from adult children enabled everyday life to carry on at home despite the increased burden of late-stage PD. This indicates a vulnerability for late-stage PD patients who do not have a spouse or other informal caregivers for support in everyday life. To ensure equal care and justice for all patients, there is a need for support for those who do not have someone to advocate their interests in contact with health care and social services.

The findings suggest that having access to PD-specialised health care is perceived as important in late-stage PD. This indicates that a specialised and multidisciplinary approach to the management of PD symptomatology is likely necessary in order to meet the different needs that exist in late-stage PD. Non-PD-specialised staff, for example, in municipality-based home health care and social services as well as in residential care facilities, should be given opportunities to obtain PD-specific education and information on a regular basis.

## Figures and Tables

**Table 1:** Participants' characteristics qualitative interview study, *n* = 20.

Participant number	Interview category	Gender	Age	HY stage	Dwelling place	Partner	Cognitive function (MMSE)	Motor function (UPDRS III)	Nonmotor symptoms (NMSS)	Depressive symptoms (GDS-30)
1	Patient	f	88	4	Home	Yes	20	39	49	21
2	Husband	—	—	—	—	Pat #1	—	—	—	—
3	Daughter	f	90	5	Residential care facility	No	20	44	147	7
4	Patient	m	83	4	Residential care facility	Yes	24	34	87	17
5	Patient	f	79	4	Home	No	24	35	46	12
6	Wife	m	73	4	Home^*∗*^	Yes	15	39	109	12
7	Patient	m	64	4	Home	Yes	19	12	106	3
8	Patient	m	65	4	Home	Yes	28	21	104	8
9	Wife	—	—	—	—	Pat #8	—	—	—	—
10	Patient	m	83	4	Home	Yes	21	48	35	8
11	Wife	—	—	—	—	Pat #10	—	—	—	—
12	Patient	m	89	4	Home	Yes	26	26	51	4
13	Wife					Pat #12				
14	Patient	m	86	4	Home	Yes	21	26	92	10
15	Wife					Pat #14				
16	Patient	m	84	5	Home	Yes	18	61	82	9
17	Wife					Pat #16				
18	Patient	m	85	4	Residential care facility	Yes	28	18	93	21
19	Patient	f	76	4	Home	Yes	22	41	51	10
20	Husband				Home	Pat #19				

Participants in chronological order. Shaded area: informal caregivers; patient demographic and clinical data are shown in cases where the informal caregivers were not part of a dyad. HY, Hoehn and Yahr staging scale (score range I–V, higher = worse); MMSE, mini-mental state examination (score range 0–30, higher = better); UPDRS, Unified PD Rating Scale, part III = motor examination (score range 0–108, higher = worse); NMSS, Nonmotor Symptoms Scale (0–360, higher = worse); GDS-30, Geriatric Depression Scale (score range 0–30, higher = worse).  ^*∗*^Patient in the process of moving to a residential care facility.

## Data Availability

Anonymized original data may be obtained at the discretion of the corresponding author upon request.
